# Completion Nephrectomy Outcomes Following Prior Partial Nephrectomy in Hereditary Kidney Cancer Syndromes

**DOI:** 10.3390/curroncol33080468

**Published:** 2026-08-06

**Authors:** Patrick D. Michael, Lauren Loebach, Ruben Blachman-Braun, Hangcheng Fu, Braden Millan, Jaskirat Saini, Sandeep Gurram, W. Marston Linehan, Mark W. Ball

**Affiliations:** Urologic Oncology Branch, National Cancer Institute, National Institutes of Health, Bethesda, MD 20892, USA; patrick.michael@nih.gov (P.D.M.);

**Keywords:** hereditary kidney cancer, completion nephrectomy, von Hippel–Lindau disease, renal cell carcinoma, partial nephrectomy, perioperative outcomes, renal function, bilateral multifocal renal cancer, hereditary renal cancer syndrome

## Abstract

Hereditary kidney cancer syndromes predispose patients to multifocal, bilateral and/or recurrent renal tumors, requiring repeated interventions over a lifetime. Despite nephron-sparing surgery being the preferred management strategy, some patients ultimately require completion nephrectomy—radical nephrectomy following prior ipsilateral partial nephrectomy—when tumor burden, declining renal function, surgical complications, or pre-transplant indications necessitate definitive kidney removal. Data on outcomes in this population remain limited. We evaluated a prospectively maintained cohort of 58 patients who underwent completion nephrectomy at the National Institutes of Health between 2000 and 2024, following at least one prior ipsilateral partial nephrectomy. Perioperative complication rates and renal functional change were the primary outcomes of interest. Von Hippel–Lindau disease was the most common hereditary diagnosis (62.1%). Open surgery was performed in 56.9% of cases. Overall complications occurred in 43.1% of patients, with 24.1% experiencing a Clavien–Dindo grade ≥ 3 event; perioperative mortality was 6.9%. The median preoperative estimated glomerular filtration rate was 72 mL/min/1.73 m^2^, declining to 50 mL/min/1.73 m^2^ at the first postoperative clinic visit. Completion nephrectomy in hereditary kidney cancer syndrome patients is associated with substantial perioperative morbidity, mortality, and expected renal functional decline. These findings inform patient counseling and surgical decision-making in this high-risk population.

## Simple Summary

Hereditary kidney cancer syndromes can cause patients to develop multiple kidney tumors throughout their lifetimes, often requiring repeated surgeries to remove tumors while preserving kidney function. When partial removal is no longer feasible due to the burden of disease, declining kidney function, surgical complications, or the need for a kidney transplant, complete kidney removal may become necessary. This procedure is technically demanding because prior surgeries leave scar tissue, altered anatomy, and distorted vasculature that increase operative complexity. This study examined outcomes in 58 patients who underwent complete kidney removal after one or more prior partial kidney operations at the National Institutes of Health, a center with specialized expertise in hereditary kidney cancer management. These procedures carry significant risks, including surgical complications and, in some cases, death. Kidney function declined after surgery but remained stable over time. These findings help patients and surgeons better understand what to expect and support informed decision-making about when and how to proceed with this challenging but occasionally necessary operation.

## 1. Introduction

Hereditary kidney cancer syndromes such as von Hippel–Lindau (VHL), Birt–Hogg–Dubé (BHD), hereditary leiomyomatosis and renal cell cancer (HLRCC), tuberous sclerosis complex (TSC), and hereditary papillary renal carcinoma (HPRC) predispose patients to multifocal and recurrent renal tumors, requiring lifelong surveillance and repeated interventions [1,2,3,4]. Over recent decades, nephron-sparing surgery has become the preferred strategy to preserve renal function and quality of life in these populations [5]. However, for some patients, cumulative tumor burden, progressive oncologic risk, and the functional toll of repeated interventions ultimately necessitate definitive surgical removal of the affected kidney [6,7,8].

Completion nephrectomy is defined as a radical nephrectomy performed in a patient with one or more prior ipsilateral surgeries and is a uniquely challenging operation, as dense perinephric fibrosis, vascular remodeling, and postoperative adhesions from prior partial nephrectomies or minimally invasive renal mass ablation increase complexity. Postoperative changes following partial nephrectomy and their impacts on subsequent nephron-sparing surgery are relatively well-understood. Reoperative partial nephrectomy is not undertaken lightly and is frequently referred to tertiary centers given its known complexity. Obliteration of tissue planes and adhesions are frequently identified as explanations for higher intra- and postoperative complication rates [9,10]. Given the more approachable nature of a radical nephrectomy, completion nephrectomy may carry less perceived stigma than pursuing repeat partial nephrectomy or ureteropelvic reconstructive surgery. Though completion nephrectomy is generally recognized by urologic oncologists as a technically difficult procedure, there remains limited literature describing functional outcomes, perioperative morbidity, and mortality in the hereditary kidney cancer syndrome population. One study described laparoscopic completion nephrectomy outcomes in the general renal cancer population of 33 patients and noted that 16 (48.5%) had a postoperative complication, 9 (27.3%) had a Clavien grade > 3 event, and open conversion was necessary in 12 (36%) [11].

To our knowledge, a comprehensive study of robotic and open completion nephrectomies has not been performed, particularly in a cohort comprised predominantly of hereditary cancer syndrome patients. Bridging this knowledge gap is essential to improve understanding of the risk factors associated with completion nephrectomy, both for providers and patients. This study was not designed to compare nephron-sparing surgery with radical nephrectomy. Rather, it reports the perioperative outcomes, renal functional changes, and complication patterns of patients who, after one or more prior ipsilateral partial nephrectomies, ultimately required completion nephrectomy. We further sought to contextualize operative morbidity, mortality, and postoperative renal function decline in this high-risk cohort, thereby informing clinical decision-making regarding the timing and counseling surrounding completion nephrectomy.

## 2. Materials and Methods

This study evaluated a prospectively maintained cohort of patients who underwent completion nephrectomy between 2000 and 2024 at the National Institutes of Health (NIH) Clinical Center. The study was conducted in accordance with the Declaration of Helsinki and approved by the Institutional Review Board of the National Institutes of Health (protocol No. NCI-97-C-0147); all participants provided written informed consent at the time of enrollment. Eligible patients had previously undergone at least one prior ipsilateral partial nephrectomy. Represented hereditary syndromes included VHL, BHD, HLRCC, TSC, HPRC, and other bilateral or multifocal hereditary phenotypes consistent with genetic predisposition. Completion nephrectomies were categorized by indication, including oncologic progression or risk, complications precluding partial nephrectomy, deteriorating unilateral renal function, or pre-transplant preparation. Procedures were performed by six urologic oncologists with expertise in hereditary renal cancer management. The surgical approach was determined at the surgeon’s discretion.

Data collected included demographics, comorbidities, operative details, postoperative complications, length of stay, transfusion requirements, and perioperative mortality. Perioperative mortality was defined as death occurring within 30 days of surgery or at any point during the index hospitalization. Renal function was assessed using estimated glomerular filtration rate (eGFR) at three timepoints: preoperatively, at postoperative nadir, and at the first postoperative follow-up visit. The primary outcomes of interest were perioperative complication rates and renal functional change. Exclusion criteria included incomplete demographic, clinical, or perioperative information related to either prior ipsilateral procedures or completion nephrectomy. Additionally, patients whose completion nephrectomy occurred during the postoperative admission for the prior partial nephrectomy were excluded. Strengthening the Reporting of Observational Studies in Epidemiology (STROBE) cohort study checklist was utilized as guidance for the development of this manuscript (Supplementary Table S1). All items on the checklist were addressed, and those items not applicable to our study were indicated as such [12].

The statistical analysis was performed using SPSS v.30. In accordance with data distribution on a normality test, continuous variables were reported as medians and interquartile ranges [IQR: 25th to 75th percentile] and compared using the Mann–Whitney U test, while categorical variables were reported as absolute values (*n*) and frequencies (%), and were compared using the chi-square test. A *p*-value < 0.05 was considered statistically significant.

## 3. Results

In the prospectively maintained cohort of hereditary and bilateral or multifocal renal cancer patients at the NIH, 58 patients met the inclusion criteria. Baseline demographic characteristics at the time of completion nephrectomy are shown in Table 1 and operative characteristics and outcomes in Table 2. Von Hippel–Lindau disease was the most prevalent syndrome (62.1%), and nine patients (15.6%) had bilateral multifocal renal cancer without a known germline variant (Supplementary Table S2). The final surgical approach was open in 56.9% of cases, and a minimally invasive approach was used in 43.1% of patients. Laparoscopy was performed in 3.4% of patients. Six (10.3%) of the intended minimally invasive cases required conversion to open surgery. Ten patients (17.2%) had a solitary kidney prior to completion nephrectomy. In addition to prior partial nephrectomy, 11 (19.0%) patients had also undergone prior ipsilateral renal mass ablation.

**Figure 1:**
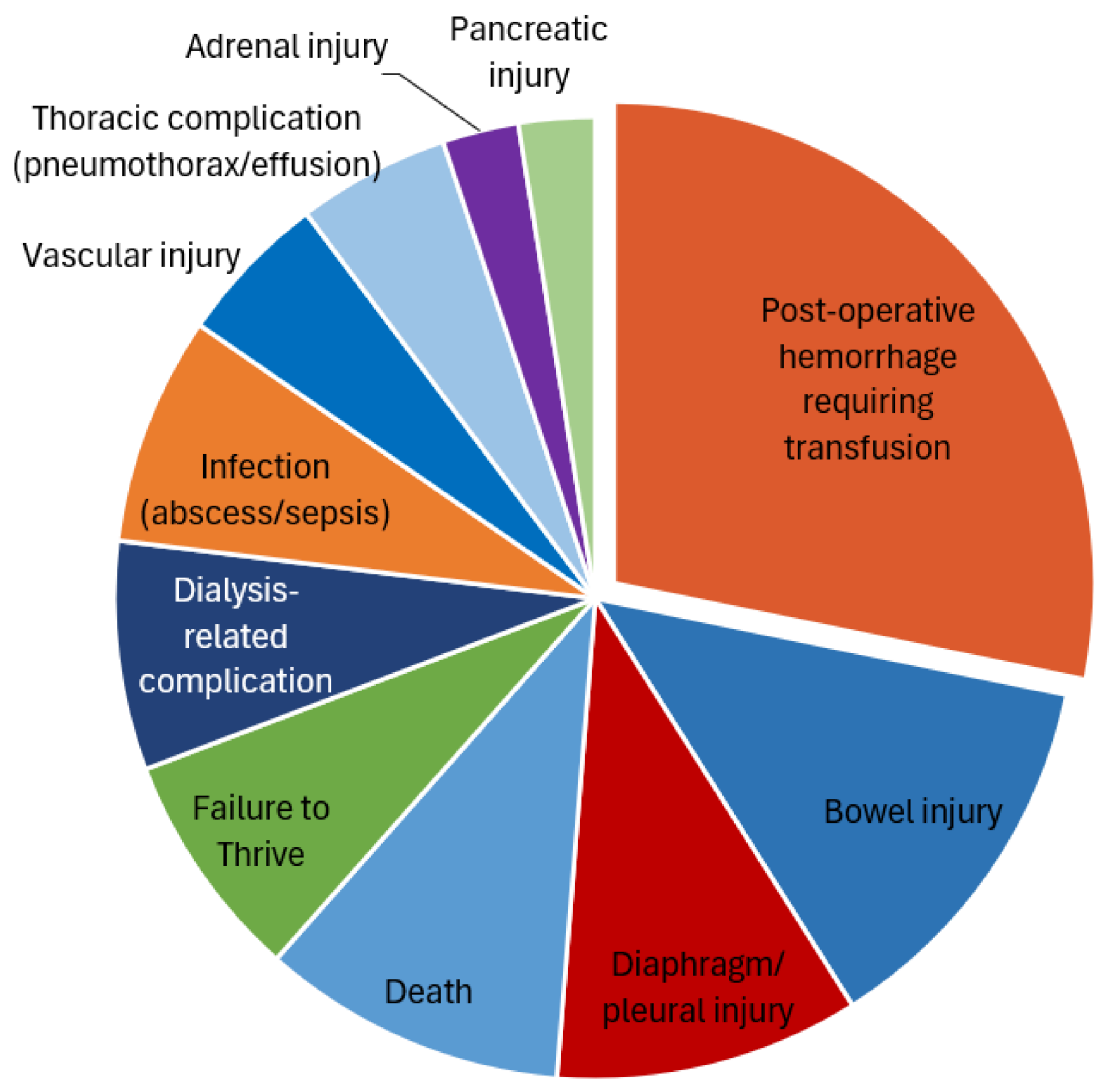
Distribution of perioperative complications among patients undergoing completion radical nephrectomy. The most common complication was postoperative hemorrhage requiring transfusion, followed by bowel injury, diaphragm/pleural injury, and death. Two patients experienced multiple complications; these patients suffered hemorrhage leading to death, and the events shown reflect the total complication rate and not unique patients.

Completion nephrectomy was performed for four principal indications: oncologic risk, progressive renal unit functional decline, pre-transplant surgical preparation, and management of complications precluding partial nephrectomy. Oncologic risk referred to patients whose tumor size or growth kinetics exceeded the threshold at which continued surveillance was felt to be safe and for whom a further partial nephrectomy was not considered safe or technically feasible. Complications that precipitated completion nephrectomy included urine leak, arterial thrombosis, infection, and significant renal scarring precluding safe enucleation and reconstruction. The number of prior partial nephrectomies per kidney ranged from one to three. The total number of tumors previously removed during prior ipsilateral partial nephrectomies (historical tumor burden) ranged from one to seventy-three, with a median of 8, emphasizing the magnitude of prior surgical intervention in this group. Twenty-five (43.1%) patients experienced postoperative complications, and 14 (24.1%) patients were classified as having a Clavien–Dindo level three complication or higher. Complications in this cohort included anemia requiring blood transfusion, bowel injury, diaphragmatic/pleural injury, sepsis, abscess, and death as seen in Figure 1.

Overall, four patients (6.9%) died during the perioperative period. One patient experienced an intraoperative vascular complication in the setting of extensive hilar adhesions and subsequently developed pulseless electrical activity (PEA) arrest. A second patient with significantly impaired baseline cardiac function underwent completion nephrectomy complicated by postoperative hemorrhage, PEA arrest, and hypoxic brain injury, with death occurring after transition to comfort-focused care. A third patient experienced PEA arrest four weeks after surgery during a prolonged postoperative hospitalization complicated by ongoing nutritional and drainage requirements. The fourth patient was discharged postoperatively but later experienced an out-of-hospital cardiopulmonary arrest and died on postoperative day 15. Although the death was suspected to be related to opioid overdose, this could not be definitively confirmed, as an autopsy was declined. All four patients died within 30 days postoperatively.

Complications and Clavien–Dindo classifications are summarized in Table 3. Clinical and surgical characteristics of patients according to the occurrence of major postoperative complications are reported in Supplementary Table S3. Baseline demographic, clinical, and perioperative characteristics were similar between groups, with no statistically significant differences in age, sex, comorbidities, preoperative renal function, surgical indication, surgical approach, prior ipsilateral renal surgery history, or solitary kidney. Patients who developed major complications (Clavien–Dindo ≥ III) had a significantly higher EBL (2775 [725–4100] mL vs. 725 [262.5–1950] mL, *p* = 0.041), whereas operative time and postoperative renal functional outcomes did not differ significantly between groups.

The distribution of postoperative complications according to solitary kidney status and the number of prior ipsilateral surgeries (1, 2, or 3 prior ipsilateral surgeries) did not significantly differ. However, when assessing the final surgical approach, the severity of postoperative complications differed significantly (*p* = 0.022), with the robotic radical approach demonstrating the highest proportion of patients without complications (82.6%), whereas open surgery was associated with a greater frequency of Grade V complications (Supplementary Table S4).

Renal function declined after surgery, as illustrated in Table 2. The median preoperative eGFR was 72 mL/min/1.73 m^2^ [IQR 46.8–94], with a wide interquartile range reflecting variability in baseline kidney function across patients with differing degrees of prior surgical intervention and chronic kidney disease. The nadir postoperative eGFR fell to 46 mL/min/1.73 m^2^. This represents a ΔeGFR of −26 [−42.5 to −5] indicating a substantial functional loss immediately following completion nephrectomy. Postoperative eGFR remained stable at the first follow-up clinic visit, which occurred at a median time of 3.7 months postoperatively, and the eGFR at this time point was 50 mL/min/1.73 m^2^ [17–67.5].

**Figure 2:**
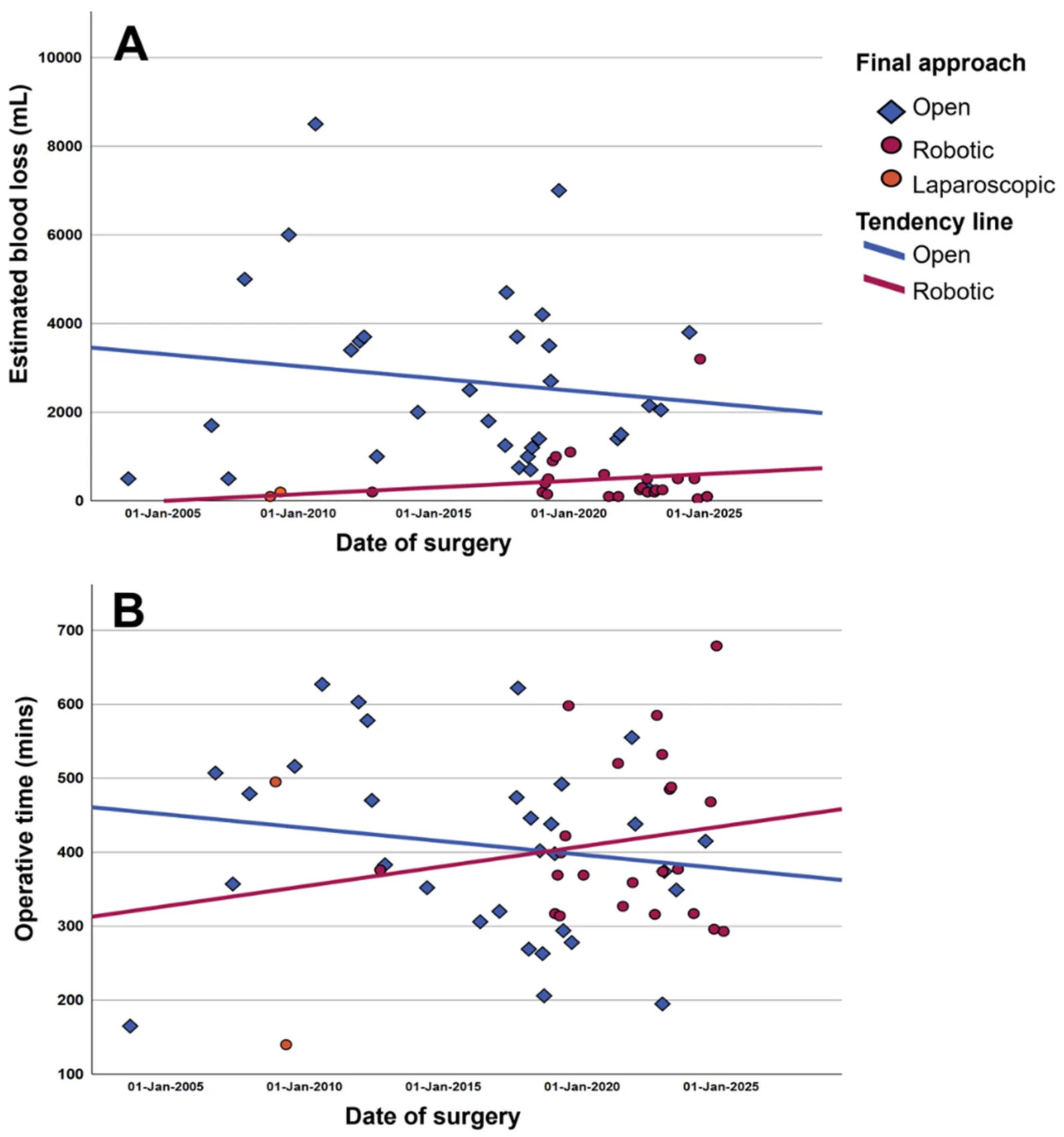
Scatterplots depicting estimated blood loss (**A**) and operative time (**B**) over the study period, stratified by surgical approach. Tendency lines for open and robotic completion nephrectomy procedures are included to illustrate temporal changes in estimated blood loss and operative time throughout the duration of the surgical cohort.

To further evaluate changes over the 24-year study period, important intraoperative variables, including EBL and operative time, were stratified by final surgical approach to assess distinct temporal trends. Specifically, the scatterplots suggested an overall tendency toward increased EBL and operative time among robotic cases, whereas open radical nephrectomy showed a tendency toward decreasing EBL and operative time over time (Figure 2).

## 4. Discussion

This study represents one of the largest cohorts examining completion nephrectomy outcomes following prior partial nephrectomy in patients with hereditary kidney cancer syndromes and bilateral multifocal kidney cancer. The findings demonstrate that completion nephrectomy in this population is associated with substantial perioperative morbidity (43.1% overall complications, 24.1% Clavien–Dindo ≥ 3), mortality (6.9%), and expected but stable declines in renal function. These outcomes reflect the unique technical challenges inherent to reoperative surgery in a field altered by prior interventions, as well as the cumulative physiologic burden of serial renal procedures in patients with hereditary syndromes.

The predominance of open surgical approaches (56.9%) in this cohort underscores the technical challenges imposed by prior ipsilateral procedures. Previous studies have demonstrated that completion nephrectomy is technically demanding, with high rates of open conversion when minimally invasive approaches are attempted. Shah et al. reported a 36% conversion rate from laparoscopic to open surgery for completion nephrectomy following partial nephrectomy, with converted cases experiencing significantly higher blood loss (725 mL vs. 175 mL), longer operative times (280 vs. 160 min), and increased major complications (58% vs. 9.5%) [11]. The technical difficulty stems from dense perinephric fibrosis, vascular remodeling, and postoperative adhesions that are particularly pronounced in this population given the number and extent of prior ipsilateral interventions. In our study, the temporal increase in EBL and operative time observed among robotic completion nephrectomies may reflect a gradual shift toward the robotic management of increasingly complex cases as institutional experience with robotic surgery matured. This finding suggests that, over the course of the study, urologists began applying the robotic approach to progressively more challenging cases.

The cumulative impact of serial partial nephrectomies has been well-documented. Antony et al. demonstrated a stepwise decline in renal function with each reoperative partial nephrectomy, with an average eGFR decline of 6.1 mL/min/1.73 m^2^ per procedure, alongside progressive increases in surgical duration, estimated blood loss, and high-grade complications [7]. In the present study, patients underwent one to three prior partial nephrectomies, with historical tumor burden ranging from one to seventy-three lesions, emphasizing the magnitude of prior surgical intervention in this cohort.

The observed perioperative mortality rate of 6.9% is substantially higher than contemporary rates for primary nephrectomy. Large population-based studies report 30-day mortality rates of 0.5–0.9% for radical nephrectomy and 0.1–0.4% for partial nephrectomy [13,14,15,16]. The elevated mortality in this cohort likely reflects multiple contributing factors: the technical complexity of reoperative surgery, comorbidities arising from multi-organ involvement due to underlying hereditary syndromes, and the physiologic stress of removing a kidney in patients who may already have compromised renal reserve. The four perioperative deaths in this cohort reflect the risks inherent to the complexity of this population, even when managed at a referral center with expertise in managing these patients.

The operative data in this cohort further underscore the exceptional complexity of completion nephrectomy after prior nephron-sparing surgery. The median estimated blood loss of 1000 mL, with an upper quartile of 2825 mL, and the median operative time of approximately 399 min—with one quarter of operations exceeding eight hours—are substantially greater than those reported for primary radical nephrectomy. These figures are not merely technical descriptors; they reflect the dense perinephric fibrosis, hilar distortion, and obliterated tissue planes that accumulate with prior ipsilateral intervention, and they translate directly into higher transfusion requirements, greater physiologic stress, and elevated conversion risk [11,13,17]. The complication profile observed—including anemia requiring transfusion, chylous ascites, pneumothorax, and bowel injury—is also consistent with the known risks of abdominal surgery in reoperative fields. From a counseling standpoint, patients considering completion nephrectomy should understand that these operations frequently entail prolonged anesthesia, a meaningful likelihood of transfusion, and a non-trivial risk of major morbidity. From a systems standpoint, the magnitude of blood loss and operative time supports a low threshold for referral of these patients to high-volume centers with the multidisciplinary surgical, anesthetic, and critical-care infrastructure required to manage them safely.

The decline in renal function following completion nephrectomy represents an expected consequence of removing a functioning kidney. This pattern of immediate postoperative decline followed by stabilization is well-established in the nephrectomy literature [18,19]. Importantly, the baseline eGFR in this cohort was already reduced compared to the general population, reflecting the cumulative functional impact of prior partial nephrectomies in the setting of an underlying hereditary syndrome. The wide range reflects the heterogeneity in baseline kidney function across patients with differing degrees of prior surgical intervention and chronic disease burden. The stabilization of renal function at follow-up, rather than progressive decline, suggests that the remaining contralateral kidney provides adequate compensatory function in most patients, though many will require close nephrology follow-up.

For patients with solitary kidneys prior to completion nephrectomy (17.2% in this cohort), the procedure commits them to renal replacement therapy, making the decision particularly consequential. Pre-transplant evaluation and coordination with nephrology and transplant services are essential for these patients [20]. The observed complications and mortality rates should inform shared decision-making and ensure that patients have realistic expectations about perioperative risks and postoperative quality of life.

The management of hereditary kidney cancer syndromes, particularly von Hippel–Lindau disease, has evolved toward nephron-sparing approaches to preserve renal function while preventing metastatic disease [21]. Current guidelines recommend intervention when the largest renal tumor reaches 3 cm in diameter, balancing oncologic control against the need to preserve renal function over a lifetime of recurrent tumors [22]. This “3 cm rule” has been validated in multiple studies showing low metastatic rates with this approach while delaying the need for more aggressive interventions [23,24]. However, despite optimal nephron-sparing strategies, some patients ultimately require completion nephrectomy. The recent approval of belzutifan, a HIF-2α inhibitor, for VHL disease-associated renal cell carcinoma represents a paradigm shift that may reduce the need for surgical interventions, including completion nephrectomy, in select patients [21,22,25]. Long-term data from the LITESPARK-004 trial demonstrated sustained tumor responses and delayed need for surgery in patients with VHL-associated renal tumors.

Each of these findings has important implications for patient counseling and surgical decision-making. Patients and clinicians must understand that completion nephrectomy represents a high-risk procedure with substantial morbidity and mortality, particularly in patients who have undergone multiple prior renal interventions. The decision to proceed with completion nephrectomy should involve multidisciplinary discussion, careful assessment of oncologic necessity versus functional preservation, and consideration of alternative strategies, including systemic therapy when appropriate.

This study has several limitations inherent to its retrospective design and the rarity of the population. Most notably, the cohort spans 24 years (2000–2024), an interval over which surgical technique, perioperative and critical care, and the systemic therapy landscape for hereditary renal cell carcinoma have all evolved substantially; the increasing adoption of minimally invasive approaches and the introduction of HIF-2α inhibition mean that outcomes accrued early in the study period may not fully reflect contemporary practice. To partially address this, we evaluated intraoperative variables over time, although the resulting subgroups are small and any temporal comparison must be interpreted cautiously. A second and more fundamental limitation is the difficulty of disentangling patient comorbidity risk factors from procedure-related complexity; such that adverse outcomes cannot be cleanly attributed to the operation itself rather than to the high-risk phenotype of the patients who require it. The heterogeneity of hereditary syndromes, indications, and prior surgical histories further limits our ability to isolate specific risk factors, and the modest number of major complications constrains statistical power. Renal functional outcomes were also limited by missing laboratory data on follow-up. Finally, this is a single-center experience from a tertiary referral center with unique expertise in hereditary kidney cancer, which limits generalizability. Long-term oncologic and quality-of-life outcomes were not comprehensively captured and represent important areas for future investigation.

## 5. Conclusions

Completion nephrectomy following one or more prior partial nephrectomies in patients with hereditary kidney cancer syndromes is a technically complex but occasionally necessary endpoint in the management of these patients. In this cohort, the procedure was associated with substantial perioperative morbidity, a perioperative mortality rate of 6.9%, and an expected decline in renal function that remained stable over follow-up. Although completion nephrectomy may ultimately be necessary, these findings emphasize the importance of specialized, multidisciplinary care and appropriate preoperative counseling, given the complexity of both these patients and these operations.

## Figures and Tables

**Table 1:** Baseline demographic and clinical characteristics of study participants. Values are presented as median [interquartile range 25th to 75th] for continuous variables and *n* (%) for categorical variables.

Demographic and Clinical Characteristics	Overall
*n*	58
Age (years)	55.5 [45.8–60.5]
Female (%)	18 (31.0%)
Race/ethnicity	
White (%)	49 (84.5%)
Black (%)	5 (8.6%)
Asian (%)	1 (1.7%)
Other, multiracial, or not reported (%)	3 (5.2%)
Diabetes mellitus (%)	11 (19.0%)
Hypertension (%)	44 (75.9%)
Preoperative Cr (mg/dL)	1.19 [0.94–1.72]
Preoperative eGFR (mL/min/1.73 m^2^)	72 [46.8–94]
Preoperative CKD stage 3b (%)	12 (20.7%)
Prior ipsilateral ablation (%)	11 (19.0%)
Prior ipsilateral partial nephrectomy	
1 (%)	39 (67.2%)
2 (%)	15 (25.9%)
3 (%)	4 (6.9%)
Solitary kidney (%)	10 (17.2%)

Cr: creatinine; eGFR: estimated glomerular filtration rate; CKD: chronic kidney disease.

**Table 2:** Operative characteristics and intraoperative and postoperative outcomes of completion nephrectomy (*N* = 58). Values are presented as median [interquartile range] for continuous variables and *n* (%) for categorical variables.

Operative Characteristics and Outcomes	
Indication for surgery	
Complication precluding partial nephrectomy (%)	6 (10.3%)
Oncologic risk (%)	37 (63.8%)
Poor function of renal unit (%)	8 (13.8%)
Pre-transplant (%)	7 (12.1%)
Time since last ipsilateral partial nephrectomy (months)	47 [25–72]
Prior ipsilateral tumor burden (tumors) *	8 [2–16]
Laterality	
Left (%)	33 (56.9%)
Right (%)	25 (43.1%)
Final approach	
Open (%)	33 (56.9%)
Robotic (%)	23 (39.7%)
Laparoscopic (%)	2 (3.4%)
Intended approach converted to open (%)	6 (10.3%)
Operative time (min)	398.5 [317–492]
EBL (mL)	1000 [287.5–2825]
Postoperative peak Cr (mg/dL)	1.79 [1.39–7.27]
Postoperative nadir eGFR (mL/min/1.73 m^2^)	46 [7–56]
ΔeGFR (mL/min/1.73 m^2^)	−26 [−42.5–−5]
eGFR (mL/min/1.73 m^2^) at first follow-up **	50 [17–67.5]

EBL: estimated blood loss; Cr: creatinine; eGFR: estimated glomerular filtration rate; ΔeGFR: change in eGFR from preoperative baseline to postoperative nadir. * Prior ipsilateral tumor burden reflects the cumulative number of tumors resected across all previous ipsilateral partial nephrectomies. ** In total, 45 patients included with a median follow-up of 3.7 [3.1–4.6] months; 4 patients died in the perioperative period; 3 patients had no documented follow-up visit, and the remaining patients did not have evaluable renal function lab work at the time of the 3-month follow-up.

**Table 3:** Categorization of postoperative complications by Clavien–Dindo grade among study participants (*N* = 58).

Clavien–Dindo Classification	Total Complications
No complications reported	33 (56.9%)
I and II	11 (19.0%)
IIIa	6 (10.3%)
IIIb	3 (5.2%)
IVa	1 (1.7%)
IVb	0 (0%)
V	4 (6.9%)

Clavien–Dindo grades: I–II = minor complications requiring no or minimal intervention; IIIa = complications requiring intervention without general anesthesia; IIIb = complications requiring intervention under general anesthesia; IVa = single organ dysfunction requiring ICU management; IVb = multiorgan dysfunction requiring ICU management; V = death.

## Data Availability

The data presented in this study are available on request from the corresponding author. The data are not publicly available due to patient privacy and institutional restrictions.

## Supplementary Materials

The following supporting information can be downloaded at https://www.mdpi.com/article/10.3390/curroncol33080468/s1: Table S1: STROBE cohort study checklist., Table S2: Distribution of reported hereditary renal cell carcinoma diagnoses among study participants and reported postoperative complications. Table S3. Clinical and surgical characteristics of the analyzed patients, with comparisons according to postoperative complications, stratified into patients with Clavien–Dindo grade ≥ III versus Clavien–Dindo grade < III and no postoperative complications. Table S4. Categorization of reported Clavien–Dindo complications in accordance with the solitary kidney status, final surgical approach, and prior ipsilateral renal surgery.

## Abbreviations

The following abbreviations are used in this manuscript:

BHD	Birt–Hogg–Dubé syndrome
CKD	Chronic kidney disease
Cr	Creatinine
EBL	Estimated blood loss
eGFR	Estimated glomerular filtration rate
HIF-2α	Hypoxia-inducible factor 2 alpha
HLRCC	Hereditary leiomyomatosis and renal cell cancer
HPRC	Hereditary papillary renal carcinoma
IVC	Inferior vena cava
NIH/NCI	National Institutes of Health/National Cancer Institute
PEA	Pulseless electrical activity
ROSC	Return of spontaneous circulation
RCC	Renal cell carcinoma
STROBE	Strengthening the Reporting of Observational Studies in Epidemiology
TSC	Tuberous sclerosis complex
VHL	Von Hippel–Lindau disease
