# Supplementary material for: Completion Nephrectomy Outcomes Following Prior Partial Nephrectomy in Hereditary Kidney Cancer Syndromes
Source: Curr Oncol. 2026 Aug 6;33(8):468. doi: 10.3390/curroncol33080468 (PMC13510471; doi:10.3390/curroncol33080468)
Supplement: Supplementary file 1 [file curroncol-33-00468-s001.zip › curroncol-4414050-supplementary.pdf]

Supplementary Table S1. STROBE cohort study checklist.

Item No.	Section/Topic	Recommendation	Location in Manuscript
**Title and Abstract**			
1a	Title and abstract	Indicate the study's design with a commonly used term in the title or the abstract	Title; Abstract
1b	Title and abstract	Provide in the abstract an informative and balanced summary of what was done and what was found	Abstract
**Introduction**			
2	Background/rationale	Explain the scientific background and rationale for the investigation being reported	Section 1 (Introduction)
3	Objectives	State specific objectives, including any prespecified hypotheses	Section 1 (Introduction), final paragraph
**Methods**			
4	Study design	Present key elements of study design early in the paper	Section 2 (Methods), first paragraph
5	Setting	Describe the setting, locations, and relevant dates, including periods of recruitment, exposure, follow-up, and data collection	Section 2 (Methods), first paragraph
6	Participants	(a) Give the eligibility criteria, and the sources and methods of selection of participants. Describe methods of follow-up; (b) For matched studies, give matching criteria and number of matched sets	Section 2 (Methods): inclusion/exclusion criteria; follow-up eGFR at first postoperative clinic visit defined; matching not applicable
7	Variables	Clearly define all outcomes, exposures, predictors, potential confounders, and effect modifiers. Give diagnostic criteria, if applicable	Section 2 (Methods): demographics, comorbidities, operative details, complications, LOS, transfusion, eGFR at three timepoints; Clavien-Dindo grading specified
8	Data sources/measurement	For each variable of interest, give sources of data and details of methods of assessment (measurement). Describe comparability of assessment methods if there is more than one group	Section 2 (Methods): prospectively maintained cohort; data elements defined
9	Bias	Describe any efforts to address potential sources of bias	Section 2 (Methods): exclusion of patients with incomplete data; Section 4 (Discussion), Limitations paragraph
10	Study size	Explain how the study size was arrived at	Section 2 (Methods): all eligible patients from prospectively maintained cohort of a rare population included; no formal sample size calculation
11	Quantitative variables	Explain how quantitative variables were handled in the analyses. If applicable, describe which groupings were chosen and why	Section 2 (Methods): continuous variables as median [IQR 25th–75th]; categorical as n (%)
12	Statistical methods	(a) Describe all statistical methods, including those used to control for confounding; (b) Describe any methods used to examine subgroups and interactions; (c) Explain how missing	Section 2 (Methods); incomplete data addressed via exclusion criteria; Table 2 footnote accounts for 45/58 patients with evaluable follow-up eGFR; Supplementary

		data were addressed; (d) If applicable, explain how loss to follow-up was addressed; (e) Describe any sensitivity analyses	
**Results**			
13	Participants	(a) Report the numbers of individuals at each stage of study—e.g., numbers potentially eligible, examined, and included in the analysis; (b) Give reasons for non-participation at each stage; (c) Consider use of a flow diagram	Section 3 (Results), first paragraph: 58 patients met inclusion criteria
14	Descriptive data	(a) Give characteristics of study participants (e.g., demographic, clinical, social) and information on exposures and potential confounders; (b) Indicate the number of participants with missing data for each variable of interest; (c) Summarise follow-up time (e.g., average and total amount)	Table 1; Table 2 footnote; Supplementary
15	Outcome data	Report numbers of outcome events or summary measures over time	Section 3 (Results); Table 2; Table 3; Figure 1; Figure 2
16	Main results	(a) Give unadjusted estimates and, if applicable, confounder-adjusted estimates and their precision (e.g., 95% confidence interval). Make clear which confounders were adjusted for and why they were included; (b) Report category boundaries when continuous variables were categorized; (c) If relevant, consider translating estimates of relative risk into absolute risk for a meaningful time period	Section 3 (Results)
17	Other analyses	Report other analyses done—e.g., analyses of subgroups and interactions, and sensitivity analyses	Supplementary
**Discussion**			
18	Key results	Summarise key results with reference to study objectives	Section 4 (Discussion), first paragraph
19	Limitations	Discuss limitations of the study, taking into account sources of potential bias or imprecision. Discuss both direction and magnitude of any potential bias	Section 4 (Discussion), Limitations paragraph
20	Interpretation	Give a cautious overall interpretation of results considering objectives, limitations, multiplicity of analyses, results from similar studies, and other relevant evidence	Section 4 (Discussion): contextualized against Shah et al. [12], Antony et al. [7], and population-based nephrectomy mortality data [14–17]
21	Generalisability	Discuss the generalisability (external validity) of the study results	Section 4 (Discussion): single-institution tertiary referral center; noted that results may not be broadly generalizable
**Other Information**			
22	Funding	Give the source of funding and the role of the funders for the present study and, if applicable, for the original study on which the present article is based	Funding section; Acknowledgments

Supplementary Table S2. Distribution of reported hereditary renal cell carcinoma diagnoses among study participants and reported postoperative complications

Reported diagnosis	Overall	Clavien-Dindo < III	Clavien-Dindo ≥ III	p-value
BHD	3 (5.2%)	3 (6.8%)	0	
HLRCC	5 (8.6%)	3 (6.8%)	2 (14.3%)	
HPRC	1 (1.7%)	1 (2.3%)	0	
TSC	4 (6.9%)	4 (9.1%)	0	
VHL	36 (62.1%)	25 (56.8%)	11 (78.6%)	
Others	9 (15.6%)	8 (18.2%)	1 (7.1%)	0.431

BHD: Birt-Hogg-Dubé Syndrome, HLRCC: Hereditary leiomyomatosis and renal cell cancer, HPRC: Hereditary Papillary Renal Carcinoma, TSC: Tuberous Sclerosis Complex, VHL: von Hippel-Lindau Disease.

Supplementary Table S3. Clinical and surgical characteristics of the analyzed patients, with comparisons according to postoperative complications, stratified into patients with Clavien–Dindo grade ≥ III versus Clavien–Dindo grade < III and no postoperative complications.

	**Overall**	**Clavien-Dindo < III or no complications**	**Clavien-Dindo ≥ III**	**p-value**
**n**	58	44	14	
**Age** (years)	55.5 [45.8 - 60.5]	55.5 [43.8 - 60]	55.5 [45.8 - 63.5]	0.649
**Female** (%)	18 (31.0%)	12 (27.3%)	6 (42.9%)	0.327
**Race/ ethnicity**				
White (%)	49 (84.5%)	36 (81.8%)	13 (92.9%)	
Black (%)	5 (8.6%)	4 (9.1%)	1 (7.1%)	
Asian (%)	1 (1.7%)	1 (2.3%)	0	
Other, multiracial, or not reported (%)	3 (5.2%)	3 (6.8%)	0	0.689
**Diabetes mellitus** (%)	11 (19.0%)	9 (20.5%)	2 (14.3%)	0.608
**Hypertension** (%)	44 (75.9%)	31 (70.5%)	13 (92.9%)	0.151
**Preoperative Cr** (mg/dL)	1.19 [0.94 - 1.72]	1.18 [0.94 - 1.69]	1.13 [0.96 - 4.46]	0.669
**Preoperative eGFR** (ml/min/1.73 m^2)	72 [46.8 - 94]	73.5 [50 - 94]	69 [28.3 - 90.5]	0.445
**Preoperative CKD stage 3b** (%)	12 (20.7%)	7 (15.9%)	5 (35.7%)	0.111
**Indication for surgery**				
Complication precluding partial nx (%)	6 (10.3%)	3 (6.8%)	3 (21.4%)	
Oncological risk (%)	37 (63.8%)	30 (68.2%)	7 (50.0%)	
Poor function of renal unit (%)	8 (13.8%)	7 (15.9%)	1 (7.1%)	
Pre-transplant (%)	7 (12.1%)	4 (9.1%)	3 (21.4%)	0.197
**Time since last ipsilateral partial nephrectomy** (months)	47 [25 - 72]	43 [25 - 67]	54.5 [27 - 149.8]	0.250
**Laterality**				
Left (%)	33 (56.9%)	28 (63.6%)	5 (35.7%)	
Right (%)	25 (43.1%)	16 (36.4%)	9 (64.3%)	0.066
**Final approach**				
Open (%)	33 (56.9%)	23 (52.3%)	10 (71.4%)	
Robotic (%)	23 (39.7%)	20 (45.5%)	3 (21.4%)	
Laparoscopic (%)	2 (3.4%)	1 (2.3%)	1 (7.1%)	0.227
**Intended approach converted to open** (%)	6 (10.3%)	5 (11.4%)	1 (7.1%)	0.652
**Prior ipsilateral ablation** (%)	11 (19.0%)	8 (18.2%)	3 (21.4%)	0.787
**Prior ipsilateral partial nephrectomy**				
1 (%)	39 (67.2%)	32 (72.7%)	7 (50.0%)	
2 (%)	15 (25.9%)	9 (20.5%)	6 (42.9%)	
3 (%)	4 (6.9%)	3 (6.8%)	1 (7.1%)	0.237
**Solitary kidney** (%)	10 (17.2%)	6 (13.6%)	4 (28.6%)	0.198
**Prior ipsilateral tumor burden** (tumors)*	8 [2 - 16]	7 [2 - 13]	15.5 [5 - 38.25]	0.095
**EBL** (mL)	1000 [287.5 - 2825]	725 [262.5 - 1950]	2775 [725 - 4100]	**0.041**
**Operative time** (mins)	398.5 [317 - 492]	380 [317.8 - 484.5]	450.5 [307.3 - 507.8]	0.490
**Postoperative peak Cr** (mg/dL)	1.79 [1.39 - 7.27]	1.65 [1.34 - 3.94]	6.51 [1.49 - 9.68]	0.162
**Postoperative nadir eGFR** (ml/min/1.73 m^2)	46 [7 - 56]	47 [19 - 58.5]	10 [6 - 53.5]	0.164
**Δ eGFR** (ml/min/1.73 m^2)	-26 [-42.5 - -5]	-26 [-42.3 - -5.5]	-25 [-59 - 2]	0.876

* Records available for 51 patients. CKD: Chronic Kidney Disease, Cr: Serum creatinine, EBL: Estimated blood loss, eGFR: Estimated glomerular filtration rate. Median [Interquartile range 25th to 75th].

Supplementary Table S4. Categorization of reported Clavien-Dindo complications in accordance with the solitary kidney status, final surgical approach, and prior ipsilateral renal surgery.

	**Solitary kidney**			**Final surgical approach**				**Prior ipsilateral surgery**			
	**No**	**Yes**	**p-value**	**Laparoscopic**	**Open**	**Robotic**	**p-value**	**1**	**2**	**3**	**p-value**
No complications reported	29 (60.4%)	4 (40%)		1 (50%)	13 (39.4%)	19 (82.6%)		24 (61.5%)	6 (40%)	3 (75%)	
I and II	9 (18.8%)	2 (20%)		0	10 (30.3%)	1 (4.3%)		8 (20.5%)	3 (20%)	0	
IIIa	3 (6.3%)	3 (30%)		0	5 (15.2%)	1 (4.3%)		1 (2.6%)	5 (33.3%)	0	
IIIb	2 (4.2%)	1 (10%)		1 (50%)	1 (3.0%)	1 (4.3%)		3 (7.7%)	0	0	
IVa	1 (2.1%)	0		0	1 (3.0%)	0		1 (2.6%)	0	0	
IVb	0	0		0	0	0		0	0	0	
V	4 (8.3%)	0	0.243	0	3 (9.1%)	1 (4.3%)	**0.022**	2 (5.1%)	1 (6.7%)	1 (25%)	0.090
